# Book Reviews

**Published:** 1799-08

**Authors:** 


### POPULAR OR DOMESTIC MEDICINE.

*A Table of Symptoms, pointing out such as distinguish one disease from another; as well as those which show the degree of danger in each disease. To which are added, Observations on the excessive indulgence of Children; particularly intended to shew its injurious effects on their Health, and the difficulties it occasions in their treatment during sickness:*—By James Parkinson.

These two supplements, though apparently distinct from each other, are intended as additional parts to the author's work, we have mentioned with merited praise in the third Number of our first Volume, p. 310, and of which we have given a farther account in the fifth Number of the same Volume, pp. 497 and 498. We have therefore nothing more to add, than our wishes, that his humane and well directed labours may be attended with that degree of public approbation, to which they appear to us fully and justly entitled.

*An Essay on the most rational means of preserving health; and of attaining to an advanced age:*—To which are added Anecdotes of Longevity. 112 pp. 12mo. Price three shillings, *London*, Wallis, 1799.

This small compilation is obviously made with a good intention; as it contains a considerable variety of useful precepts, the authorities for which are carefully mentioned. Although it contain neither original, nor *always punctually applicable* rules and reflections, yet the whole is interspersed with much entertaining and dietetic information.

---

*Medicine preservative et curative, &c.—On the prevention and cure of diseases by medicines; or, a treatise on health and the practice of medicine: for the use of young practitioners, and every person regardful of health.* By Nicolas Francois Rougnon. 2 vols. 8vo. 500 pp. *Besancon*, Couché; and *Paris*, Maquignon.

Although we are long accustomed to see alluring title-pages, used by many authors with a similar intention, as the sagacious inn-keeper exposes a handsomely painted sign to attract the attention of passengers; yet in this instance, we are obliged to make an honourable exception. The aged Professor communicates to the world, in this work, the fruit of his observation and experience during fifty years medical practice; a circumstance, which seldom occurs in the modern annals of medicine.

In treating of each disorder, the author has followed the most natural division, according to the different organs and parts of the body, the derangement or affection of which produces the disease. He concisely enumerates the means of prevention, in regard to the patient's situation, temperament, age, sex, &c. It is an additional merit in the author of this treatise, that he, unlike many writers of the present day, gives only such *facts*, under the head of *practical truths*, as have borne the test of time and experience.

